# Reporting adverse events—Swedish Registered Nurses experience in a municipal home care context

**DOI:** 10.1002/nop2.223

**Published:** 2018-12-03

**Authors:** Margaretha Norell Pejner, Annica Kihlgren

**Affiliations:** ^1^ School of Social and Health Sciences Halmstad University Halmstad Sweden; ^2^ Örebro University School of Health Sciences Örebro Sweden

**Keywords:** adverse event reports, home care, risk analysis

## Abstract

**Aim:**

To describe how Registered Nurses in a municipal home care context experience adverse event reports.

**Design:**

A qualitative design was used.

**Method:**

Twelve semistructured individual interviews with Registered Nurses in a municipal home care context were collected on two occasions and analysed with qualitative content analysis.

**Results:**

The results show that conflicts exist between being able to trust the managers and their feedback, being loyal to colleagues and retaining professional pride. These are described in the theme “Contradiction” and the three categories: “Awareness”; “Uncertainty”; and “Concealment.”

## 1 INTRODUCTION

Safe delivery of care in a fast‐growing and complex healthcare system is one of the greatest challenges today. A Swedish and an international report (Socialstyrelsen, 2018a; WHO, 2017a) show that around one in ten hospitalized patients experiences some kind of harm. This harm can be related to incorrect diagnosis, treatment or medication; delayed care; or an organizational structure where the different healthcare organizations do not fully communicate with each other about the individual patient's condition or treatment. It appears that 47.7 million adverse events occur annually among hospitalized patients worldwide (WHO, 2017a). These events are estimated to cost trillions of US dollars each year due to loss of productivity and the ability of the patient and their relatives to work (WHO, 2017a). There is ongoing international work to decrease adverse events. A definition of adverse event is an event or circumstance that could have or did lead to unintended and/or unnecessary harm to a person (Socialstyrelsen, 2018b; WHO, 2005). One way to increase knowledge about risk management is through the reporting of adverse events, and many healthcare facilities have systems in place for such reporting. Unfortunately, reporting is sporadic and analyses and evaluations of the reports are poor (WHO, 2017b).

## 2 BACKGROUND

The Swedish National Board of Health and Welfare gives guidelines for patient safety (Socialstyrelsen, 2018a), which are directed to the line managers and staff. One of these guidelines addresses the importance of creating a culture of safety in the organization. This is accomplishable by having an approach where people do not blame each other, where the staff members can feel secure reporting adverse events and by having organizations that learn from adverse events. A systematic review that included 38 articles from studies conducted in 13 countries concluded that the organization has an impact on the propensity to make reports (Vrdnjak, Denieffe, O'Gorman, & Pajnkihar, 2016). According to the review, organizational barriers related to the reporting of medication errors were related to the number of patients the nurse was responsible for and the specialization of the unit. Nurses in paediatric specialization units and nurses looking after fewer patients were more prone to report events than nurses in adult units and nurses who had a greater number of patients. Additionally, a good relationship between the nurses and physicians also proved to be a significant factor that led to making reports. Other factors that affected the willingness to report events were good reporting systems and high standards of care. It was also reported that personal and professional barriers concerned fear, negligence, attitude, level of education and training. A Norwegian study conducted by Windsvold Prang and Jelsness‐ Jørgensen (2014) that investigated the barriers to reporting adverse events in the nursing home context reported similar results.

The number of persons aged 60 years and older is growing worldwide. The average age of the population is also increasing, but there is little evidence that their health is better than the health of their parents (WHO, 2017c). The development of a sustainable long‐term care system is needed to meet the growing population of older people and the numbers of older persons wanting to remain at home despite disease and/or decreased functionality (Harrefors, Sävenstedt, & Axelsson, 2009; Torgé, 2014). In Sweden, older people can remain at home despite the need for care (Motion, 1998/99: So436; SFS, 2001:453). A common organizational solution for home care is one that has the physicians, Registered Nurses (RNs) and other home care staff based in different entities. County councils or private companies employ the physicians, while municipalities employ the RNs and the other home care staff, all with different spheres of activities. All contribute in cooperation together in the care of the older persons that is regulated by law (SOSFS, 2005:27).

A study concerning the experiences of RNs working in a municipal palliative care context revealed that the organization gave RNs limited legitimacy in their own organization and in dealings with other organizations and professionals (Törnquist, Andersson, & Edberg, 2013). RNs described unclear responsibilities and cooperation issues with the physicians in the other organization about patient medication. This often led to temporary solutions or the need to involve additional persons and other professionals. An additional difficulty is that RNs due to organizational issues feel compelled to delegate the administration of the medication as well as other healthcare tasks, for example, wound care, blood drawing or blood pressure taking to unlicensed staff (Bittner & Gravlin, 2010; Craftman, Grape, Ringnell, & Westerbotn, 2016). Legislation controls the delegation of nursing care and entails a legal commitment (SOSFS, 1997:14). To ensure a safe outcome, the delegation of nursing care requires close cooperation and personal knowledge between the healthcare staff member who is the donator of the delegation and the one who is the receiver of the delegation (Bittner & Gravlin, 2010). According to Törnquist et al. (2013), this is difficult to achieve in an organization that has the various professionals coming from different healthcare entities. Despite this, in Sweden, delegated unlicensed homecare staff perform a reported 68% of the municipal elder care nursing interventions (Norell, Ziegert, & Kihlgren, 2013). Sears, Ross Baker, Barnsley, and Short (2013) concluded that the incidence of adverse events in the home care context was 66.5%, with the most common being falls and adverse drug events. In the home care context, informal caregivers who are not formally delegated nursing interventions, but are performing them, are also involved in the adverse events, which most commonly involve medication administration (Parand, Garfield, Vincent, & Franklin, 2016).

To summarize, there appears to be a lower propensity to report adverse events involving adult patients. In organizations, where there is close contact between the healthcare members, the RNs are more inclined to report adverse events (Vrdnjak et al., 2016; Winsvold Prang & Jelsness‐J ørgensen, 2014). In Sweden, the organization of the care of older persons is complex with several caregivers based in different entities (Motion, 1998/99: So436; SFS, 2001:453; SOSFS, 2005:27). Healthcare interventions are commonly delegated to unlicensed staff (Norell et al., 2013) or are performed by informal caregivers. In both cases, it is known that adverse events occur (Parand et al., 2016; Sears et al., 2013). RNs working in the municipal elderly care organization report that they have limited legitimacy, unclear responsibilities and problematic cooperation with other healthcare professionals (Törnquist et al., 2013). To prevent risks in the care of older people, it is important that adverse events be reported with the aim to learn from them (WHO, 2017b), but how do RNs in this complex organization stand about the reporting of adverse events?

### 2.1 Aim

The aim of the study was to describe how Registered Nurses in a municipal home care context experience adverse event reports.

### 2.2 Design

The study has a qualitative approach with a descriptive design.

## 3 METHOD

A qualitative study aims to describe a phenomenon as it exists, based on human life and experiences (Polit & Beck, 2008). Semistructured individual interviews were performed and analysed with qualitative content analysis since the purpose was to describe and interpret the meaning of municipal home care RNs' experiences of adverse event reports (Graneheim & Lundman, 2004).

### 3.1 Sample and recruitment

The study was conducted in a medium‐sized municipality in east central Sweden on two occasions. The inclusion criteria were RNs working in municipal home care with at least 2 years of professional experience. On both occasions, contact was made with the head managers in the municipality who gave permission for the study. Written information about the study's purpose and an invitation to participate was sent to the two unit managers recommended by the head managers. After the unit managers gave their consent, they emailed to the researchers a list of the RNs working on the day, evening and night shifts that fulfilled the inclusion criteria. A written invitation containing information about the study's purpose, approach and confidentiality was sent to 22 nurses, twelve on the first occasion and ten on the second. Telephone or e‐mail contact was then made to ask if they wanted to participate in the study. Eight RNs declined due to heavy workloads and two nurses stated they did not feel they had worked long enough in the municipality and therefore lacked enough experience with adverse event reports. Additionally, one RN had been on sick leave for a long time and therefore declined and one respondent gave no reason for not participating.

### 3.2 Data collection

The time and location of the interviews were decided with the participants via telephone. Eleven chose to have the interviews at their workplace, and one chose to have it take place in another municipality. The semistructured interview guide used at both occasions had a list of areas and issues about the topics the author wanted to highlight. The questions asked were as follows: “What are your experiences of how the adverse events were managed at your workplace?”; “What are your experiences of the support given between colleagues when an event has occurred?”; and “What are your experiences of the support given by the managers when an adverse event has occurred?” With this list as support, the interviewer encouraged the participants to speak freely (Polit & Beck, 2008). In the semistructured interview, the interviewer did not pose a large number of key questions but followed up the participant's responses with comments and prompting questions (prompts and probes). The questions were complementary and subordinate and were only used when needed to lead the participant into the intended topic (Gillham, 2008; Polit & Beck, 2008). The interviews that lasted 35–60 min were recorded and transcribed verbatim by a research secretary. The transcribed interviews were then compared with the tape recording and corrected if needed to ensure accuracy (Kvale & Brinkman, 2014).

### 3.3 Data analysis

The authors read the interviews several times to create an overall picture. Domains were identified, and the text was divided into meaning units that were condensed and abstracted during the analysis process. Codes were found, and categories were created. The categories were combined into one overall theme (Graneheim & Lundman, 2004). (Table 1.)

**Table 1:** Overview of categories and subcategories developed from the interviews

Meaning units	Condensed meaning units	Codes	Subcategories	Categories
I stand alone in my decisions and in a vulnerable situation. I mean that it is advanced. A lot can happen. You have to know what you are doing. I mean something can easily happen	Alone in the decision when something can easily happen in an advanced situation where an adverse event report can be made	Complex situations in which adverse events can happen and reports made	Complexity of tasks	Awareness
Most people are supportive but it became so clear in this case. One can show a bit of consideration for each other. It's not just this, the same attitude exists with smaller things	To give support and consideration to each other	Support and respect	Loyalty and peer support	
In such situations I think even the management is getting better and supportive, but in something this small you don't know what to do	The management does not give support in every situation	Have no trust in management	Distrust of management	Uncertainty
Of course, there are discussions. And if it is from this that has happened or that it's just for now	It is discussed, but there are ambiguities in the procedures	Unclear procedures	Lack of guidelines	
If I were to have an adverse event report written about me, I would be irritated. I would be ashamed and not discuss it with a colleague	Experience of irritation and shame	Irritation and shame	Shame	Concealment
It is stressful to write an adverse event report on a colleague	Do not write an adverse event report on a colleague	Unwritten adverse event report	Under‐reporting	

### 3.4 Ethical approval

The first data collection was made in conjunction with a master's thesis, and since the study did not collect or handle any sensitive personal data, ethical approval was not required according to the Swedish Act concerning the Ethical Review of Research Involving Humans (SFS 2003:460). Nevertheless, this part followed the standard principles and guidelines for ethical research and the head manager at each unit approved the study. Furthermore, written and oral consent was obtained from each participant. The second data collection was made in conjunction with another project where the Uppsala Regional Ethical Review Board granted Ethical approval (registration number 2013/523).

## 4 RESULTS

From the initial 22 eligible RNs, twelve agreed to participate, six on the first occasion and six on the second. They worked on the day, evening and night shifts. The participants were aged between 38–63 years. The number of years working as an RN varied from 5–41 years. The number of years employed in municipal home care was between 5 and 18 years. Ten of the participants also had community health education and were certified public health nurses. All participants were female.

**Figure 1:**
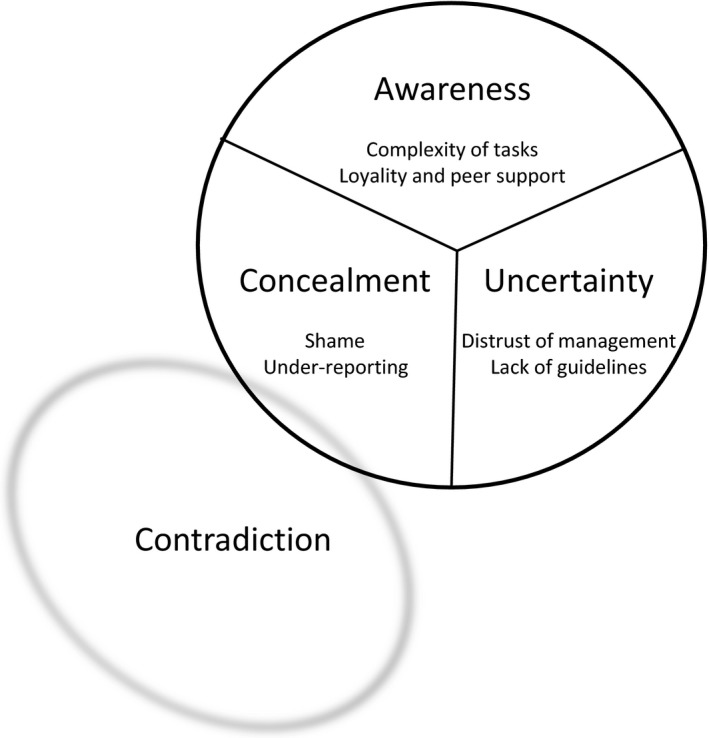
The three categories that create the theme Contradiction

The results illustrate how RNs working in municipal home care experience the reports of adverse event. Feedback, trust of management, loyalty to colleagues and professional pride are in conflict with, as described in the three categories: “Awareness”; “Uncertainty”; and “Concealment” (Figure 1). The categories and their associated subcategories create the theme “Contradiction”:

Yes, sometimes I've seen that the medication was not dispensed correctly and I've fixed it myself. Sometimes I said something afterwards and… I haven't seen that person again after that. It happens. (3)

### 4.1 Awareness

The RNs reported that they like their job especially since they are not bound to one specific type of care and have the opportunity to meet many different patients and their relatives, as well as staff from other occupational categories. This offers them an overall perspective of the patient's situation, which is something they considered important. At the same time, they were aware that their work situation is negatively affected by how it is organized. RNs are not always present to make assessments because the patients are cared for in their homes. Interventions are often decided based on information usually acquired from nurse assistants. The RNs worked alone with high workloads and a large number of home visits. They reported that the stressfulness of this situation increases the risk of adverse events. Despite the fact that their work situation exposed them to increased risks, the RNs considered it their duty to report adverse events and that their managers encouraged them to do so:

We are required and we should report all the mistakes and encourage the writing of reports even the mistakes made by those in other occupational categories that are pertinent to us (5)

#### 4.1.1 Complexity of tasks

The RNs experienced that their tasks have become increasingly more complex. Developments in medical technology have led to changes, and patients who would have remained hospitalized a few years ago are now being discharged with continued care at home. Previously, the tasks consisted of basic nursing interventions, for example, dispensing of medication, simpler wound care or blood pressure monitoring. Multimorbidity among patients today makes them more vulnerable. Their condition demands increased supervision, more complex interventions involving technical equipment, polypharmacy and more advanced nursing interventions:

They have rather serious illnesses. And it's not only one; they have multiple illnesses that intertwine with each other. (12)

The home environment is not easily adapted to this kind of care, which contributes to additional risk factors and subsequent adverse events. The RNs expressed their concerns that things could go wrong and discussed their situations and conduct about the adverse event reports with their fellow colleagues.

#### 4.1.2 Loyalty and peer support

All of the RNs described how they could talk to each other about their concerns over working in an environment where adverse event reports would need to be written by them or about them. When they felt uneasy about something or unsure of some new procedure, they would seek information and work together until they felt secure enough to work independently. The same applied to those occasions when someone made an error:

We help each other and that is an incredible support. (10)

The RNs expressed that there is an understanding that something can go wrong and were empathetic with colleagues that did something incorrectly. Despite this loyalty and peer support that they had for each other, they also expressed a desire to have the adverse event reports discussed formally at their workplace meetings.

### 4.2 Uncertainty

The RNs were aware of the risks for error in their work and welcomed formal discussion of the reports, but they experienced uncertainty over the consequences the reports might bring from the management. Some RNs had the opinion that the managerial personnel were not involved in the RNs' daily routines and therefore did not have an understanding of how it all actually functions:

I get the feeling that… if it should happen to me, I don't think I would get much support. (1)

The RNs told how they desired emotional, but mainly legal support from their managers. Emotionally, they needed support managing their feelings associated with having a report written that involved them, or for having written an inaccurate report. They needed legal support because of a possible legal process where they could lose their license. RNs also had the opinion that their managers needed to be more involved in the discussions of the adverse event reports and that it must be made clearer the kind of events that should be reported.

#### 4.2.1 Distrust of management

The RNs were uncertain over the management's reaction when they reported adverse events. Some believed that those having a good relationship with the management would receive better support than those who did not and some would get none at all. Further uncertainty existed over which instances support would be offered. They were concerned that the management might not offer support with all of the adverse events, but only when they involved something rather serious:

When big things like that happened, then there is automatic support from the medically responsible nurse and the National Board of Health. Then it's an entirely different ball game. (5)

Not knowing how or even if the management would react, caused the RNs to feel insecure about the adverse event reports. At times, they would have wanted a discussion with their managers about the reports, but felt their managers had little understanding of their situation, as they were not working close to them or involved in their daily work.

#### 4.2.2 Lack of guidelines

RNs described how the lack of clarity and routines about adverse events and what they are, created insecurity. At times, they were unsure when or even if an adverse event report should be written. An RN explained that she only reported adverse events when it was someone else that had been involved. Other RNs explained how when they had made the mistake, they only made a note about it in the patient records:

Some even write adverse event reports on…if the medication is not taken even though it is perhaps the patient himself that manages it, you understand…? (6)

All of the RNs expressed a desire for strict routines and that the routines should be the same everywhere. They also wanted some form of risk assessment in relation to the adverse events that had occurred.

### 4.3 Concealment

Even though RNs understood that the purpose of reporting adverse events was to promote safe practices and procedures, it was described as a sensitive subject. Telling a colleague that something wrong occurred, was not something they liked to do. The RNs it seems would rather avoid addressing the adverse event:

This with adverse event reporting and then you [the RN] need to make it clear that… you didn't betray someone if you write one. I think it's that that many of us [RNs] feel, that a person feels a little….that it's just not fun to write one. (1)

Adjusting incorrectly dispensed medications was common, but they reported that they would rather do that than discuss it with their colleagues. When an adverse event was discussed with a colleague, they would often conclude that it was not so serious after all because nothing bad had happened. Both the person who brought up the subject and the one responsible for the adverse event experienced a sense of shame.

#### 4.3.1 Shame

RNs described that reporting adverse events that involved colleagues was distressing. When they did, it was after repeated errors and several discussions with the concerned person. It was easier to report adverse events when it was the hospital at fault, as the hospital is an entity rather than individual that was to blame. If an adverse event report was made and a person did not know about it until afterwards, it could be experienced as irritating. At the same time, the RNs also described that it could be insulting to remind someone of something they did wrong. The RNs who had been responsible for an adverse event described it as annoying, but they were also ashamed and preferred not to discuss it with their colleagues:

First I didn't say anything because I was ashamed. Then I thought; are there others that have experienced this? Then I was ready to talk about it. I know there are things done incorrectly that people do not talk about. It's sensitive. (2)

#### 4.3.2 Under‐reporting

The RNs explained that adverse events were not reported to the extent that they should have been. Reporting a colleague was rarely done. Additionally, when the RN did not know, the circumstances behind an adverse event the RN might not report it. Moreover, they told how time was limited and writing reports could take too much time. Despite this, they expressed a desire for a more open and comprehensive attitude towards adverse event reports:

I think we should talk more about the things that go wrong. Perhaps at our workplace meetings. That this has happened to this person or myself and what can we do to avoid it. (4)

## 5 DISCUSSION

### 5.1 Methodological considerations

The purpose of the study was to describe the experience of the phenomenon; how RNs in a municipal home care context experienced adverse event reports. Using a qualitative design with content analysis according to Graneheim and Lundman (2004) gives the opportunity to explore this area, but some questions must be discussed. Collecting data using individual interviews provides for a deeper understanding (Polit & Beck, 2008), but collecting data on two different occasions can be seen as a limitation. Since only six RNs were involved in the initial data collection in a master's thesis, additional data were collected as a part of another project. That project focused on decision‐making among municipal home care nurses when the health of the older persons deteriorated. Adverse events were affecting the RNs when decisions needed to be made, which is why the researchers decided to interview them about their experiences of adverse event reports. Whether the questions about two related topics have affected their response is difficult to know. By merging data from two different occasions, an opportunity existed to obtain a better sample size and achieve greater depth in the analysis. It also gave added variation in age, education and experience. The participants gave rich descriptions of their experiences of the phenomena that were studied. According to Sandelowski (1995), the sample size in qualitative research should be large enough to achieve a variation of experiences and small enough to permit a deep analysis of the data. To increase credibility, the process from selection and collection to analysis and results has been clarified in the text and quotations have been included. The analysis has been discussed in the author's research groups. The authors have experience in municipal home care, which can be seen as both a strength and a limitation during the interviews and the analysis of the data. Experience in the field provides insight into ask questions that can give new knowledge or confirm existing knowledge in the field, but it increases the risk of unconscious interpretations (Polit & Beck 2008). To minimize unconscious interpretations, the second author has not been involved in the data collection. Through a critical, reflective approach during the interviews, the authors have attempted to minimize this risk. Working conditions in municipal organizations in Sweden are similar; therefore, the results can be considered transferable nationally, but not necessarily internationally.

### 5.2 Result discussion

The resulting theme *“Contradiction”* illustrates how RNs in a municipal home care context experience adverse event reports. This study shows that RNs on the one hand have good knowledge and peer support about risk situations in their daily work and the subsequent consequences. On the other hand, they assumed a reserved position towards management and their colleagues, which results in an under‐reporting of adverse events. The RNs in this study have experienced that the transition from hospital care to home care coupled with short hospital stays can create vulnerable situations for the patients. Moreover, technology has led to more advanced treatments and interventions in the home care setting. The organization of the care dictates the involvement of several caregivers, which seldom provides RNs with the opportunity to make their own assessments and causes them to rely on the opinions of other caregivers or relatives. The Swedish National Board of Health and Welfare (Socialstyrelsen, 2018b) claims that the home environment is not easily adapted to the advanced care that is thought to be offered in the home. The home environment is not adaptable to the hygienic, occupational or technical standards for care, and it also involves different actors that can be difficult to coordinate. There seems to be an awareness and some dialogue between the authorities about the difficulties and risks for adverse events associated with this kind of organization (Socialstyrelsen, 2018b). At the same time, the Swedish government advocates (Motion, 1998/99: So436; SFS, 2001:453) the individual's right to remain at home if they need care for an illness, or if they are receiving end of life care. One can see that there is not only a feeling of contradiction among the Registered Nurses in this study, but also an overall contradiction between the individual's rights and the ability to deliver safe care. Even if there is an awareness of the difficulties and risk situations in the home care sector, currently, there is no real debate about its organization. However, it seems that RNs in this study use an approach of not blaming each other to manage adverse events and this is in line with one of the Swedish national guidelines (Socialstyrelsen, 2018b) for risk management. They state that they are supporting each other with the more risk‐filled practices and procedures when an adverse event has occurred. From this point of view, it seems that they are actively working to promote a safe culture and prevent adverse events. The second and the third guidelines involve the managers who should create the conditions for the culture of safety and adverse event reporting. Staff members' sense of security and trust together with clear routines and guidelines are considered fundamental for the culture of safety (Socialstyrelsen, 2018b). This study revealed that the RNs were unsure how their managers would act after an adverse event was reported. The importance of management in municipal home care is a recurrent topic in the literature (Josefsson & Hansson, 2011; Larsson Kihlgren, Fagerberg, Skovdahl, & Kihlgren, 2003; Winsvold Prang & Jelsness‐J ørgensen, 2014). The authors describe that RNs in the municipal home care setting have experienced that their managers have a lack of knowledge about the different tasks involved in their daily work, which creates uncertainty in their decision‐making. Josefsson and Hansson (2011) also conclude that this could be the reason for unsolved conflicts between RNs and their managers. Barriers to adverse event reporting are believed to stem from deficiencies in leadership (Winsvold Prang & Jelsness‐J ørgensen, 2014). Deficiencies such as lack of managerial feedback and non‐updated guidelines and routines were also cited by the RNs in this study. Boamah, Spence Laschinger, Wong, and Clarke (2017)emphasize the importance of leadership that plays in the achievement of good outcomes and conclude that a transformational leadership increases patient safety outcomes. Transformational leadership is characterized by the relationship between the manager and the staff (Bass, 1985). Managers gain a trust through their presence that motivates the staff in their work. Sexton et al. (2018) also emphasize the presence of the manager and conclude that the culture of patient safety and staff satisfaction increase when the manager is present in the daily work. RNs in this study expressed a distrust for their managers that were absent in their daily work. In the guidelines for patient safety (Socialstyrelsen, 2018b), the managers are provided with directions on how they and their staff members can work, with an emphasis on working together as a team. It would be interesting to investigate the manager's role in relation to patient safety.

The results in this study show that RNs are aware of the risks involved in their daily work and that adverse events should be reported. They talk with one another and support each other, but they are also ashamed of being involved in an adverse event. Healy (2012) comments in a reflective article that RNs are often reminded that they could lose their license, which creates a culture of fear and shame.

A study conducted by Eun‐Ho (2016) describes how RNs in South Korea perceive their professional status. The study consisted of 31 RNs that described their professional status as falling into one of three categories, “I am proud of my nursing job” (twelve answers), “I am not proud of my nursing job” (nine answers) and “Advocating for change and improvement” (ten answers). It would be interesting to see if there is a correlation between those three categories and the inclination to report adverse events. If that is the case, further work should be done to strengthen the culture of safety to decrease adverse events in the healthcare sector.

## 6 CONCLUSION

RNs in a municipal home care context describe how there is contradiction in their experiences of adverse event reports. On the one hand, is their awareness of the different risk situations involved in their daily work that they could talk about and the support they have for each other in the event of an adverse event. Despite their awareness and peer support about adverse events, it appears that adverse events are under‐reported. This is related to the RNs being ashamed of being involved in an adverse event. On the other hand, they were uncertain of their managers. The uncertainty lies in how their managers will act towards them in case they are involved in an adverse event. RNs also complained that the guidelines are often diffuse or missing. In a continued effort to minimize risks and adverse events in a home care context, closer cooperation is required between RNs and the management to improve the reporting of adverse events and achieve clarity and security in the RNs' daily work.

## CONFLICT OF INTEREST

No conflict of interest has been declared by the authors.
